# “Especially for People Our Age”: Exploring the Correlates of Ageism During the COVID-19 Pandemic

**DOI:** 10.1093/geroni/igab046.2292

**Published:** 2021-12-17

**Authors:** Jennifer Sublett, Michael Vale, Toni Bisconti

**Affiliations:** 1 The University of Akron, Akron, Ohio, United States; 2 University of Akron, Akron, Ohio, United States

## Abstract

The COVID-19 pandemic has presented an unprecedented context for older adults where they may feel patronized, isolated, and fearful because of their greater risk of getting COVID-19 and being targets of ageism. Previous researchers have linked ageism negatively with health and well-being; although, the majority of this research has highlighted the negative, or hostile, aspects of ageism, and excluded the overaccommodative and patronizing qualities of benevolent ageism. Since the start of the pandemic, both forms of ageism have been noted to be more salient with claims of an ageism outbreak (Ayalon et al., 2020). The correlates of ageism during the COVID-19 pandemic are widely unknown, and the goal of this study was to explore whether experiences of ageism were related to different affective and health-related responses to the pandemic. In a sample of older adults (N=65) collected in September 2020, we found that benevolent ageism positively correlated with pandemic specific experiences of pity (r=.27, p<.05), loneliness (r=.30, p<.05), worry (r=.40, p<.01), and negatively related to self-reported physical health (r=-.31, p<.05) and emotional well-being (r=-.26, p<.05). Hostile ageism did not relate to pity, but positively correlated with loneliness (r=.25, p<.05) and worry (r=.37, p<.01), and negatively related to physical health (r=-.27, p<.05) and emotional well-being (r=-.38, p<.01). This work provides preliminary evidence of how the lives of older adults have been influenced by COVID-19 and the resulting ageism outbreak. Future research should continue this avenue of study with more expansive and inclusive samples and approaches as the pandemic is not over.

